# Comparative profiling of miRNA expression in developing seeds of high linoleic and high oleic safflower (*Carthamus tinctorius* L.) plants

**DOI:** 10.3389/fpls.2013.00489

**Published:** 2013-12-02

**Authors:** Shijiang Cao, Qian-Hao Zhu, Wanxia Shen, Xiaoming Jiao, Xiaochun Zhao, Ming-Bo Wang, Lixia Liu, Surinder P. Singh, Qing Liu

**Affiliations:** ^1^Commonwealth Scientific and Industrial Research Organization Plant IndustryACT, Australia; ^2^National Citrus Engineering Research Center, Citrus Research Institute, Southwest UniversityChongqing, China; ^3^National Key Laboratory of Plant Genomics, Institute of Microbiology, Chinese Academy of SciencesBeijing, China; ^4^School of Life Sciences, Northeast Normal UniversityChangchun, China

**Keywords:** miRNA profiling, safflower, *Carthamus tinctorius*, high oleic, comparative analysis

## Abstract

Vegetable oils high in oleic acid are considered to be advantageous because of their better nutritional value and potential industrial applications. The oleic acid content in the classic safflower oil is normally 10–15% while a natural mutant (*ol*) accumulates elevated oleic acid up to 70% in seed oil. As a part of our investigation into the molecular features of the high oleic (HO) trait in safflower we have profiled the microRNA (miRNA) populations in developing safflower seeds expressing the *ol* allele in comparison to the wild type high linoleic (HL) safflower using deep sequencing technology. The small RNA populations of the mid-maturity developing embryos of homozygous *ol* HO and wild type HL safflower had a very similar size distribution pattern, however, only ~16.5% of the unique small RNAs were overlapping in these two genotypes. From these two small RNA populations we have found 55 known miRNAs and identified two candidate novel miRNA families to be likely unique to the developing safflower seeds. Target genes with conserved as well as novel functions were predicted for the conserved miRNAs. We have also identified 13 miRNAs differentially expressed between the HO and HL safflower genotypes. The results may lay a foundation for unraveling the miRNA-mediated molecular processes that regulate oleic acid accumulation in the HO safflower mutant and developmental processes in safflower embryos in general.

## Introduction

microRNAs (miRNAs) are small endogenous non-coding RNAs of ~21 nucleotides (nt) in length and have been identified in animal, plant, virus and a single-celled eukaryote (Bartel, 2004; Jones-Rhoades et al., 2006). It is well established that miRNA expression across species is highly regulated in a time-dependent and tissue-specific manner (Lagos-Quintana et al., 2002; Babak et al., 2004; Druz et al., 2012). In higher plants, miRNAs have been clearly shown to regulate a number of developmental and physiological processes by mediating target gene silencing at transcriptional and/or post-transcriptional levels (Bartel, 2004; Wu et al., 2010). Some miRNA targets are themselves regulators, such as those encoding transcription factors and F-box proteins (Bartel, 2004; Jones-Rhoades et al., 2006; Mallory and Vaucheret, 2006). In higher plants, primary miRNA transcripts (pri-miRNAs) are processed by RNase III-like enzyme DICER-LIKE 1 (DCL1) resulting in a short imperfect stem-loop precursor (pre-miRNA) that is further cleaved by DCL1 to release a miRNA/miRNA^*^ duplex. The mature miRNA strand is incorporated into the RNA-induced silencing complex (RISC) to recognize its target(s), whereas the miRNA^*^ strand is subject to degradation (Bartel, 2004; Sunkar and Zhu, 2004).

Plant miRNAs have been identified by two approaches including direct cloning (Sunkar and Zhu, 2004; Wang et al., 2004; Lu et al., 2005; Sunkar et al., 2005) and computational prediction followed by experimental validation (Jones-Rhoades and Bartel, 2004; Zhang et al., 2005; Gleave et al., 2008). Recently developed high-throughput sequencing technologies have markedly contributed to the expanding knowledge of small RNA (sRNA) in eukaryotic cells through discovery of a number of newly evolved and species-specific miRNAs (Rajagopalan et al., 2006; Fahlgren et al., 2007, 2009; Pantaleo et al., 2010). This strategy has been widely applied to many plants for miRNA identification (Fahlgren et al., 2007; Moxon et al., 2008; Sunkar et al., 2008). The criteria for miRNA characterization and annotation in plants have been reported (Meyers et al., 2008), i.e., miRNA precursors should be able to form a stem-loop structure, and mature miRNAs should be detected by RNA Northern blot analysis, RT-PCR or sequencing (Ambros et al., 2003). Sequence complementarity between miRNAs and their target genes is very high in higher plants, which has made the search for plant miRNA target genes a relatively straightforward process (Jones-Rhoades and Bartel, 2004).

Safflower (*Carthamus tinctorius* L.) is an ancient oilseed crop that is currently grown for its high quality edible oil and bird seed. Oleic acid and linoleic acid are the two major fatty acids in safflower oil, the relative proportion of which largely determines the oil's nutritional value and functional properties (Knowles, 1989). Both oleic acid and linoleic acid can lower total serum cholesterols, but oleic acid has markedly higher oxidative stability than linoleic acid as it contains one fewer reactive double bond. The oxidatively stable high oleic (HO) safflower oil not requiring hydrogenation that can result in the formation of nutritionally undesirable *trans* fatty acids is increasingly appreciated in the edible oil markets (Ascherio et al., 1999; Mozaffarian et al., 2006). Beyond food applications, HO vegetable oils also have significant existing and potential industrial uses in biodiesel, lubricants, and hydraulic oils because of the high oxidative stability required in these products. The original HO trait in safflower, found in an introduction from India, was controlled by a partially recessive allele *ol* at a single locus *OL* (Knowles and Hill, 1964; Knowles, 1989). Oleic acid content of *olol* genotype is usually 71–75% of total fatty acids (Knowles, 1989). The *ol* allele has now been incorporated into safflower breeding program worldwide and has resulted in the release of numerous HO safflower varieties including Saffola 317 (S-317) that was used in the current study.

More recently, identification of miRNAs from safflower mature seed, leaf, and petal by high-throughput sequencing has been reported (Li et al., 2011a). However, the expression patterns of miRNAs in safflower developing seeds during the active period of lipid metabolism still remain unknown. In this study we report the deep sequencing and comparative analysis of sRNAs in the HO and conventional high linoleic (HL) safflowers. At least 55 previously described miRNAs, together with two candidate novel miRNA families, were identified in both HL and HO miRNA pools, by aligning sRNAs to the mature miRNAs in miRBase v18 and using the Mireap software developed by BGI (Beijing Genome Institute, Shenzhen, China). Further, the expression of a few selected previously known miRNAs was confirmed by stem-loop reverse transcription polymerase chain reaction (RT-PCR) and/or Northern blot analysis. Potential target genes that were implicated in a wide range of biological processes including transcription regulation and metabolism were predicted for the known and novel safflower miRNAs. The differential expression of miRNAs in the HL and HO safflower genotypes was also examined. Understanding the miRNA expression pattern and identification of novel miRNAs in developing safflower seeds of both the HO and HL genotypes may shed light on the role of miRNA in seed development and oil metabolism.

## Materials and methods

### Plant materials and growth conditions

Safflower (*Carthamus tinctorius* L.) plants of HO genotype S-317 and HL wild type SU were grown from seed in a perlite and sandy loam potting mix in greenhouse under a day/night cycle of 16 h (25°C)/8 h (22°C). S-317 was supplied by Devexco International, and SU was a common bird seed safflower that was obtained from Heffernan Seeds in NSW, Australia. Developing seeds were harvested at 15 days post-anthesis (DPA), when the seed development reaches to mid-maturity and seed mass increases and oil accumulation become the most rapidly (Hill and Knowles, 1968).

### RNA isolation and small RNA sequencing

Small RNAs were extracted from safflower developing seeds using the mirVana™ miRNA Isolation Kit (Ambion, California, USA) following the manufacturer's instruction. Isolated small RNAs were quantified and assessed for quality using a Nanodrop ND-1000 spectrophotometer (Thermo Fisher Scientific, Victoria, Australia). Then small RNAs were subjected to 15% (w/v) denaturing polyacrylamide gel electrophoresis (PAGE), and 18–25 bp portions were excised from the gel and purified. The purified small RNA molecules were then ligated to the Illumina 5′ and 3′ adaptor sequentially and converted to cDNA by RT-PCR following the Illumina protocol. Finally, PCR products were sequenced by the Beijing Genomics Institute (BGI, Shenzhen, China) using Illumina HiSeq™ 2000 Sequencing System.

### Primary analyses of the deep sequencing datasets

The raw reads were processed to remove those that were formed by adaptor-adaptor ligation, low quality and less than 18-nt in length. The remaining reads were used in the following up analyses. The Compositae Genome Project (CGP) safflower expressed sequence tag (EST) database (http://cgpdb.ucdavis.edu/asteraceae_assembly/data_assembly_files/GB_ESTs_Feb_2007.sp.Cart_tinc.clean.assembly) was used to identify reads mapped to the safflower genome and potential pre-miRNAs using the SOAP program v1.11. The small RNAs were also compared with the sequences of non-coding RNAs available in Rfam (http://www.sanger.ac.uk/software/Rfam) and the GenBank (http://www.ncbi.nlm.nih.gov/) to classify tRNA, rRNA, snoRNA and snRNA.

### Identification of known miRNAs and prediction of novel miRNAs

After removing small RNAs aligned to above mentioned known non-coding RNAs, the remaining high quality reads were first searched against all mature miRNA sequences deposited in miRBase v18 (http://www.mirbase.org/blog/2011/11/mirbase-18-released/) to identify safflower miRNAs conserved in other plant species, and then aligned to the CGP safflower ESTs (http://compgenomics.ucdavis.edu/compositae_data.php?name=Carthamus+tinctorius) to identify precursors of known safflower miRNAs. A small RNA was considered as a candidate known miRNA if it has ≤2 mismatches and/or ≤1 gap, or indel, with a known miRNA. To identify novel miRNAs, the remaining unmatched reads were first aligned to the safflower ESTs to identify those perfectly matched sequences. For the fully matched ESTs, 150 bp sequences flanking the matched small RNA were extracted and subjected to hairpin structure prediction using the Mireap program developed by BGI. The small RNAs with predicted structures that met the previously described criteria (Allen et al., 2005; Meyers et al., 2008) were retained as candidate novel miRNAs.

### Small RNA northern blot hybridization

Small RNA gel blot analysis was carried out following the previously reported procedures (Wang et al., 2008). Approximately 10 μ g of total RNAs from 15 DPA developing seeds of the HL and HO genotypes were separated in a 17% denaturing polyacrylamide gel and blotted onto Hybond-N^+^ membranes (GE Healthcare, Buckinghamshire, UK). The membranes were UV cross-linked and pre-hybridized at 42°C for 3 h in hybridization buffer containing 50% formamide, 5x SSPE (3 M NaCl, 0.2 M NaH_2_PO_4_, and 0.02 M EDTA, pH7.4), 5x Denhardt's solution (2% Ficoll 400, 2% polyvinylpyrrolidone and 2% BSA), 1 mM EDTA, 1% BSA, and 1% SDS. DNA oligos antisense to the corresponding miRNAs were end labeled by the forward reaction using 10 units of T_4_ polynucleotide kinase (Roche Molecular Biochemicals, Indianapolis, IN, USA) with the supplied buffer, to which 300 nM [γ−^32^P] ATP (3000 Ci/mmol) was added. The reaction was incubated for 1 h at 37°C. Following the removal of the unincorporated ^32^P-label using G-25 microcolumns (GE Healthcare), the radioactive probe was denatured by boiling for 5 min prior to addition into the hybridization buffer and hybridization was allowed to proceed at 42°C overnight. The membranes were then washed twice, 30 min each in 2× SSC, 0.2% SDS at 40°C. Hybridization signal was detected and analyzed by a Fujifilm FLA-5000 phosphorimager (Fujifilm, Tokyo, Japan).

### Expression analysis of miRNA by stem-loop RT-PCR

Stem-loop RT-PCR was used to detect miRNAs following previously reported procedures (Chen et al., 2005; Varkonyi-Gasic et al., 2007). Four hundred ng of low molecular weight RNAs were reverse transcribed using SuperScript III Reverse transcriptase (Invitrogen, Carlsbad, CA, US) by a pulse reverse transcription program. Pulse RT-PCR was performed at 16°C for 30 min, followed by 60 cycles at 30°C for 30 s, 42°C for 30 s and 50°C for 1 s and then 85°C for 5 min to inactivate the reverse transcriptase. One μL of RT product was used for end-point PCR. The reaction conditions were as follows: an initial polymerase activation step for 2 min at 94°C, followed by 40 cycles of 94°C for 15 s, and 60°C for 1 min. The electrophoresis of the PCR products was performed on a 4% agarose gel for 30 min under 100 voltage. The primers used to carry out stem-loop RT-PCR were listed in Supplementary Table 1.

## Results

### Analysis of the sRNA transcriptomes of the HL and HO safflower genotypes

**Figure 1:**
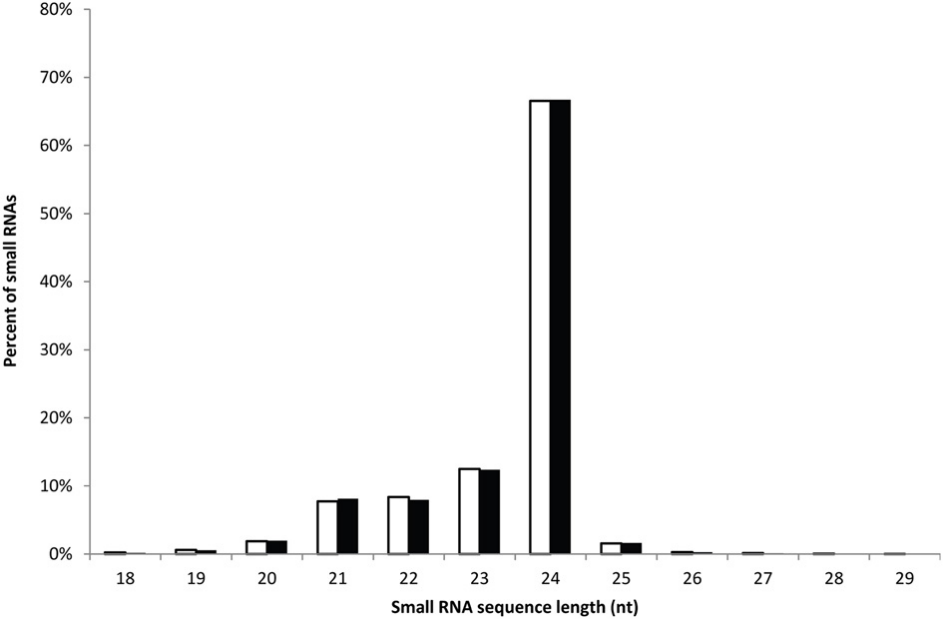
**The length distribution of safflower sRNAs**. Representation of sequences with different lengths in safflower HL and HO sRNA population is shown here. The number of sequences is expressed as a percentage of the total number of sequences.

The high-throughput Illumina sequencing technology was employed to sequence the sRNA populations of safflower developing seeds derived from a HO variety S-317 and a HL variety SU. After initial processing (e.g., trimming adaptor sequences and removing reads with polyA sequence), 22,860,098 and 21,427,392 clean reads of 18–30 nt in length were retained in the HL and HO library, respectively. These reads represent 9,674,480 and 9,029,394 unique sequences in the HL and HO genotypes, respectively. The overall size distribution pattern of sRNAs was similar between the HL and HO genotypes (Figure 1), with the majority being 21–24 nt in length, which is the typical size range for Dicer derived products (Provost et al., 2002). The 24-nt sRNAs were the most abundant in both libraries, accounting for 66.5% and 66.7% of the total number of sRNAs of the HL and HO genotypes, respectively. The sRNAs were further classified into different RNA categories. Blast searching against the GenBank and Rfam databases revealed that 0.34% of the HL and 0.33% of the HO unique sRNAs matched to annotated house-keeping non-coding RNAs, including rRNA, tRNA, snRNA and snoRNAs. A search against miRBase v18 found that 0.62 and 0.64% of the unique sRNAs in the HL and HO genotypes matched with the known miRNAs, respectively (Table 1). Since safflower genome sequence is not yet available, we aligned these sRNAs against the available safflower ESTs that were generated by CGP. This analysis showed that 224,902 of the HL and 202,624 of the HO unique sRNA sequences could be mapped to 7666 and 7551 ESTs in the HL and HO genotypes, respectively. Of the unique sRNAs mapped to ESTs, only 16.47% were found in both the HL and HO libraries whereas the remaining were found in only either the HL or the HO library (Table 2).

**Table 1:** **Analysis of the small RNA populations in HL and HO safflower developing seeds**.

**Category**	**Total reads**		**Unique reads**	
	**HL (%)**	**HO (%)**	**HL (%)**	**HO (%)**
rRNA	272,329 (1.19)	250,742 (1.17)	25,712 (0.27)	22,864 (0.25)
snRNA	4,804 (0.02)	6,369 (0.03)	1,391 (0.01)	1,410 (0.02)
snoRNA	1,551 (0.01)	1,406 (0.01)	889 (0.01)	848 (0.01)
tRNA	71,792 (0.31)	61,017 (0.28)	4,719 (0.05)	4,660 (0.05)
miRNA	848,808 (3.71)	755,876 (3.53)	59,747 (0.62)	57,603 (0.64)
other sRNAs	21,660,814 (94.75)	20,351,982 (94.98)	9,582,022 (99.04)	8,942,009 (99.03)
Total	22,860,098 (100)	21,427,392 (100)	9,674,480 (100)	9,029,394 (100)

**Table 2:** **A summary of the common and specific sRNAs in HL and HO safflower developing seeds**.

**Class**	**Unique sRNAs**	**Total sRNAs**
Common	2,644,651 (16.47)	28,581,605 (64.54)
HL-specific	7,029,829 (43.77)	8,323,222 (18.79)
HO-specific	6,384,743 (39.76)	7,382,663 (16.67)
Total	16,059,223 (100)	44,287,490 (100)

### Identification of known miRNAs

To investigate the scope of known miRNAs in safflower, we compared the unique sRNA sequences (20–24 nt) with all the mature miRNAs deposited in miRBase v18. Using the criteria of no more than two mismatches and one indel between the query sRNA sequence and a known miRNA, we identified 55 known miRNA families in our datasets (Table 3). Of these miRNAs, only two (miR156 and miR160) had corresponding pre-miRNAs in the safflower EST database (Supplementary Figure S1). This is probably because of either size limitation of EST database, quick turn-over of pre-miRNAs or low expression levels of these identified known miRNAs.

**Table 3:** **The known miRNAs identified in developing safflower seeds and their predicted targets**.

**miRNA**	**Sequence**	**Length**	**HL count^a^**	**HO count^a^**	**Fold-change log^2^(HO/HL)^b^**	**Predicted targets^c^**
cti-miR156	TGACAGAAGAGAGTGAGCAC	20	8256.4	8889.9	0.11	EL410179
cti-miR157	TTGACAGAAGATAGAGAGCAC	21	37.1	28.5	−0.38	EL374499
cti-miR159	TTTGGATTGAAGGGAGCTCTA	21	26.8	24.0	−0.16	EL389677
cti-miR160	TGCCTGGCTCCCTGTATGCCA	21	10.9	7.3	−0.57	–
cti-miR162	TCGATAAACCTCTGCATCCAG	21	6.0	5.7	−0.07	EL386787
cti-miR164	TGGAGAAGCAGGGTACGTGCA	21	120.7	104.6	−0.21	EL374434
cti-miR165	TCGGACCAGGCTTCATCCCC	20	5.8	6.0	0.06	EL390889
cti-miR166	TCGGACCAGGCTTCATTCCCCC	22	6155.2	6737.1	0.13	EL390889
cti-miR167	TGAAGCTGCCAGCATGATCTAA	22	1442.4	1735.6	0.27	–
cti-miR168	TCGCTTGGTGCAGGTCGGGAA	21	689.4	415.0	−0.73	–
cti-miR169	CAGCCAAGGATGACTTGCCGA	21	1.8	1.5	−0.25	–
cti-miR171	TGATTGAGCCGTGCCAATATC	21	34.2	52.1	0.61	–
cti-miR172	AGAATCTTGATGATGCTGCAT	21	2.9	2.7	−0.09	EL403681
cti-miR319	TTGGACTGAAGGGAGCTCCCT	21	1.9	1.3	−0.53	–
cti-miR390	AAGCTCAGGAGGGATAGCGCC	21	44.9	34.8	−0.37	–
cti-miR395	CTGAAGTGTTTGGGGGAACTC	21	1.0	16.8	4.12^**^	EL373143
cti-miR396	TTCCACGGCTTTCTTGAACTG	21	0.1	0.9	3.65	EL392642
cti-miR397	ATTGAGTGCAGCGTTGATGAA	21	21.3	81.4	1.93^**^	EL391150
cti-miR403	TTAGATTCACGCACAAACTCG	21	11.9	21.1	0.83	–
cti-miR408	TGCACTGCCTCTTCCCTGGCT	21	170.6	155.4	−0.13	EL396941
cti-miR834	TGGTAGCTGTAGAGGTGGTAGA	22	77.2	63.8	−0.27	EL398160
cti-miR845	ACAGCTCTGATACCAGTTGATA	22	7.9	7.8	−0.01	–
cti-miR858	TTCGTTGTCTGTTCGACCTTG	21	1.7	1.3	−0.47	–
cti-miR1507	CCTCGTTCCATACATCATCTAG	22	45.6	1.7	−4.72^**^	–
cti-miR1511	AACCAGGCTCTGATACCATGA	21	6.0	7.3	0.28	–
cti-miR1520	TCATCAGAGGATGACACGTGACA	23	539.8	468.3	−0.21	–
cti-miR1852	ATATAGATTCAGATTGCAGGTA	22	2.9	0.2	−3.97^**^	–
cti-miR1861	TGACTTGATGCATAAACTGAG	21	3.3	0.4	−3.14^**^	–
cti-miR1863	AGCTCTGATACCATGTTAGATTAT	24	205.9	419.5	1.03^**^	–
cti-miR2089	TTACCTATTCCTCCCATTCCA	21	2.4	1.6	−0.58	–
cti-miR2675	CGTGGATATTGGCAGGGATT	20	1.0	1.0	−0.04	EL406669
cti-miR2911	GGCCGGGGGACGGACTGGGAA	21	692.0	181.6	−1.93^**^	–
cti-miR2948	TGTGGGAGAGTTGGGCAAGAAT	22	0.1	1.1	3.03^**^	–
cti-miR2950	TGGTGTGCAGGGGGTGGAATA	21	15.5	12.6	−0.31	–
cti-miR3476	TGAAACTGAGTTTGTTGGCCGC	22	3.3	1.8	−0.91	–
cti-miR3954	ATGGACAGAGAAATCACGGTCG	22	4.1	3.5	−0.24	–
cti-miR4345	TAAGACGGAATAACACAGATT	21	1.6	3.3	1.07^**^	–
cti-miR4348	AAACTGTGTAAGATGGTGACATT	23	4.3	4.9	0.16	–
cti-miR4372	TAAAATCGTGACATGTGACAATC	23	8.9	14.5	0.70	–
cti-miR4414	AGCTGCTGACTCGTTGGTTCA	21	10.8	4.7	−1.19^**^	–
cti-miR5021	GGAAGAAGACGAAGAAGAAAA	21	8.2	6.8	−0.28	EL406158
cti-miR5026	ACTCTCTAAGATCTTGACACGT	22	0.8	626.7	9.64^**^	EL510105
cti-miR5059	CGGTCCTGGGCAGCAACACCA	21	3.0	2.6	−0.19	–
cti-miR5072	CGTTCCCCAGCAGAGTCGCCA	21	7.8	7.3	−0.10	–
cti-miR5081	TAATTTGTAGAATAATTGATGGT	23	1.4	2.8	0.98	–
cti-miR5234	TTTTATTGTGGATGGCAGAAGG	22	3.5	3.3	−0.08	–
cti-miR5290	AAGAGGAGAGAGATAGACACATA	23	84.3	73.0	−0.21	EL388536
cti-miR5291	GATGGATGGATGGATGGATGGAT	23	12.0	11.7	−0.04	EL385719
cti-miR5485	TGACAAGTTGGTATCAGAGCAA	22	5.7	2.9	−0.99	–
cti-miR5490	TTGGATTGTTTATTTAAGATGG	22	8.6	0.2	−5.53^**^	–
cti-miR5492	AGTAGGAGGATAGATAGGTT	20	17.2	14.1	−0.28	–
cti-miR5513	TAAGAAATGGACAAGAGACTGA	22	0.3	1.7	2.50^**^	–
cti-miR5523	TGGGGAGGAACATACTTACTAGT	23	1.0	1.0	−0.04	EL394147
cti-miR5628	GAAAGAGCGAAAGATATGTTTA	22	5.7	7.2	0.34	–
cti-miR5634	AGGGACTTTTTGACTTTACGGG	22	23.3	21.3	−0.13	–

a *Counts were normalized into TPM (Tags Per Million)*.

b** *p ≤ 0.01*.

c *For each miRNA, only the most confidently predicted target is shown*.

**Figure 2:**
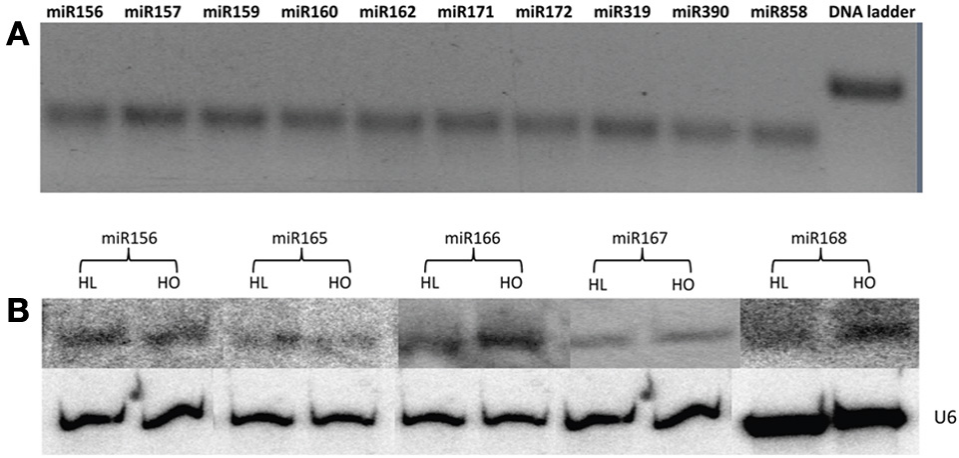
**Stem-loop RT-PCR and sRNA Northern blot analysis. (A)** Stem-loop RT-PCR to detect known miRNAs in safflower wild type. **(B)** Northern blot to examine the known miRNAs in safflower HL and HO genotypes.

Twenty-one miRNA families are conserved in both monocots and dicots (Jones-Rhoades et al., 2006), of which 17 were identified in safflower developing seeds in this study. Expression of some of these conserved miRNAs was further verified by stem loop RT-PCR and/or Northern blot analysis (Figure 2). This result confirmed conservation of these miRNAs in safflower and suggest that they are probably important for safflower seed development and metabolism. The four conserved miRNA families, for which an exact matching mature miRNA sequence was not found in this study, were miR393, miR394, miR398, and miR399. This is not surprising because at least three of these miRNAs are stress responsive and are barely expressed in Arabidopsis plants grown under normal conditions. For example, miR393 was up-regulated under salt stress conditions (Jones-Rhoades and Bartel, 2004), and miR398 has been shown to be induced under copper-deprived conditions (Sunkar et al., 2006). The expression of miR399 could not be detected in plants grown on medium containing optimal levels of phosphate, but was induced when the phosphate level was depleted in the medium (Fujii et al., 2005). The remaining candidate known miRNAs present in our safflower sRNA data, including miR403, miR858, miR1507, miR2948, miR2950, miR4414, and miR5139, all had exact matches with the published miRNA sequences from other plant species. The expression of miR858 was further confirmed by stem-loop RT-PCR (Figure 2). Other candidate known miRNAs identified in both the HL and HO safflower developing seeds had at least one mismatch or indel compared with the published mature miRNAs (Table 3). Since a pre-miRNA was not identified for any of these candidate known miRNAs in the safflower CGP EST database, their miRNA identities remain to be further verified.

A miRNA functions to regulate the expression of its specific target gene(s). Putative targets of the known safflower miRNAs were predicted based on CGP safflower ESTs using miRU (Zhang, 2005). At least one target gene each was predicted for 11 of the 17 conserved miRNA families identified in safflower developing seeds, while none for the remaining 6 conserved miRNA families (Table 3 and Supplementary Table S2). It has been established that conserved miRNAs usually have conserved targets in different plant species. That is also the case in safflower, as exemplified by miR165/166 targeting genes encoding homeobox-leucine zipper protein, miR172 targeting AP2-like transcription factor and miR397 targeting genes encoding L-ascorbate oxidase. However, some conserved miRNAs have new putative targets predicted in safflower, such as kinesin family member C2/C3, a candidate target of miR172. Of the remaining 35 candidate known miRNA families identified in safflower, only seven showed predicted targets in the CGP EST database (Table 3). Interestingly, the putative safflower miR5021 (cti-miR5021) is 24 nt in length and has a large number of predicted targets. This is in contrast to Arabidopsis miR5021 that is 20 nt in length and without a predicted target (Borges et al., 2011). Although the identity of cti-miR5021 needs further confirmation, the length of this miRNA and its large number of predicted targets suggest that it could be involved in epigenetic gene regulation through DNA methylation as it was reported for rice miR1863, rather than mRNA cleavage (Wu et al., 2010).

### Identification of novel miRNAs

**Figure 3:**
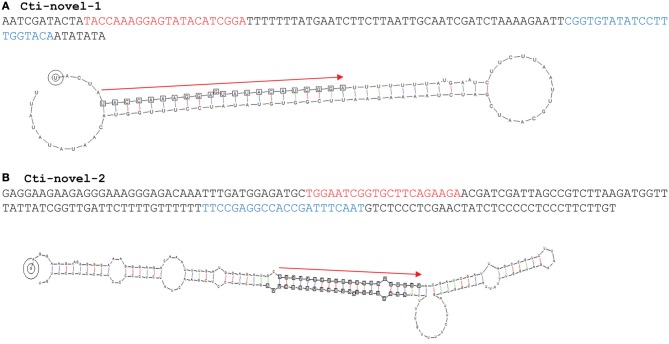
**The hairpin structures of novel miRNAs**. The precursors of the two novel miRNAs (listed in Table 4) identified in this study are shown. The miRNA and miRNA^*^ are highlighted in red and light blue, respectively. sRNAs generated from the precursor are shown only for pre-cti-novel-2. Red arrows indicate the position of miRNAs in the pre-miRNA hairpins.

A criterion that supports miRNA annotation is the identification of a stem-loop precursor from which the duplex of miRNA/miRNA^*^ is excised. Analysis using such a criterion identified two novel *MIRNAs*, identity of which was strongly supported by the presence of miRNA^*^ sequences and absence of sRNA generated from the antisense strand (Table 4; Figure 3). Similar to most conserved miRNAs, these two novel miRNAs begin with a 5′ uridine, which is a characteristic feature of many miRNAs. A putative target was predicted for cti-novel-1, but this gene has not been annotated and its function remains unknown. The read number ratio between miRNA^*^ and miRNA for cti-novel-2 was 49.9% (Figure 3), which is significantly higher than that for cti-miR156 (0.004–0.08%) and cti-miR162 (0%), the two safflower miRNAs with their pre-miRNAs identified. It is understood that the miRNA^*^ is usually quickly degraded after the miRNA/miRNA^*^ duplex is loaded into the RISC. Therefore, it will be of interest to know whether the mismatches between miRNA and miRNA^*^ at the end of miRNA (or at the beginning of miRNA^*^) is a factor affecting the degradation rate of miRNA^*^.

**Table 4:** **Novel miRNAs identified in developing safflower seeds**.

**miRNA**	**Mature miRNA sequence**	**Length**	**Precursor**	**MFES^a^**	**HL count^b^**	**HO count^b^**	**Predictedtargets**
cti-novel-1	UACCAAAGGAGUAUACAUCGGA	22	CART_TINC.CSA1.1088	35.0	766.0	567.9	EL386907
cti-novel-2	UGGAAUCGGUGCUUCAGAAGA	21	CART_TINC.CSA1.826	25.1	22.4	32.5	–

a *MFES: minimum free energy*.

b *Counts were normalized into TPM (Tags Per Million)*.

### Differential expression of miRNAs in HL and HO safflower genotypes

It has been demonstrated that high-throughput sequencing provides a viable approach to estimate the expression profiles of miRNAs. The abundance of individual miRNAs in the sRNA library could serve as an index for the estimation of their relative expression levels. Of the 55 known and two novel miRNAs, 13 were differentially expressed between the HL and HO genotypes (Tables 3, 4). The expression levels of miR395, miR397, miR1863, and miR5026 were significantly higher in the HO than in the HL genotype, whereas the expression levels of miR1507 and miR2911 were significantly lower in the HO than in the HL genotype (Table 3). Previous studies indicated that miR395 targets ATP sulfurylase genes and plays an important role in sulfate homeostasis through regulating sulphate uptake, transport and assimilation in Arabidopsis (Liang et al., 2010). miR397 has been shown to target the transcripts for copper protein plantacyanin and members of the laccase copper protein family in Arabidopsis (Abdel-Ghany and Pilon, 2008). miR1863 has been demonstrated to be required for silencing heterochromatin by methylation in rice (Wu et al., 2010). miR1507 could be involved in regulating the expression levels of NBS-LRR type disease resistance genes (Zhai et al., 2011). Whether the differential expression of these miRNAs can be attributed to oleic acid accumulation and related oil metabolism warrants further investigation.

## Discussion

miRNAs have emerged as a new class of regulatory factors and attracted much attention during the past decade. Safflower is an important oilseed crop grown for edible oil production and understanding the functions of miRNAs in regulating oil accumulation in the developing embryos of safflower could be of great value for the development of safflower germplasm with enhanced oil production and improved nutritional value.

In this work, we have characterized the sRNA transcriptomes of a HO mutant (*ol* allele) and the HL wild type safflower. In total, ~23 and ~21 million of high-quality sRNA sequences were generated from the HL and HO mid-maturity developing seeds, respectively. As in other higher plants, 21–24 nt sRNAs dominated the sRNA transcriptome in safflower with the 24-nt class being the most abundant in both the HL and HO libraries. Molecules of 24-nt processed by DCL3 are often the most abundant endogenous plant sRNAs (Vaucheret, 2006), but this may vary among species. For example, 24-nt sRNAs are the most abundant in Arabidopsis, rice, sweet orange and tomato (Rajagopalan et al., 2006; Morin et al., 2008; Moxon et al., 2008; Zhu et al., 2008; Song et al., 2010; Xu et al., 2010), whereas 21-nt sRNAs are the most abundant in grapevine, wheat and conifers (Yao et al., 2007; Dolgosheina et al., 2008; Pantaleo et al., 2010). This size distribution pattern may also be spatially regulated. For instance, Arabidopsis inflorescence showed particularly high representation of 24-nt small interfering RNAs (siRNAs) in comparison to the leaf and seedling-derived small RNAs that have increased representation of the 21-nt size class (Kasschau et al., 2007).

Deep sequencing technology has proven to be a powerful tool for characterization of sRNA population and for identification of novel miRNAs. A number of conserved miRNAs and 13 novel miRNAs have been previously identified in safflower mature seed, leaf and petal (Li et al., 2011a). We noticed that the four conserved miRNAs (miR393, miR394, miR398, and miR399), for which an exactly matched safflower sRNA was not found in our study, were reported by Li et al. (2011a) but their sequences also did not exactly match their counterparts in other plant species. In addition, their expression was very low in mature seed compared to most other conserved miRNAs (Li et al., 2011a). In this study, two more novel miRNAs were identified in immature safflower seeds, suggesting that the number of miRNAs to be identified have yet to be saturated in safflower. Therefore, it is reasonable to anticipate that additional novel miRNAs could be identified from the sRNA datasets derived from developing safflower seeds reported in this study when the safflower genome sequence becomes available in the future.

In agreement with previous reports in developing seeds of peanut (Chi et al., 2011), maize (Kang et al., 2012) and soybean (Shamimuzzaman and Vodkin, 2012), in this study we found that numerous highly conserved miRNAs, particularly miR156, miR166, miR167, and miR168, were highly expressed in safflower developing seeds. This may indicate a potential functional role of these conserved miRNAs in seed development and metabolism in safflower. Interestingly, this was also the case in mature safflower seeds (Li et al., 2011a), suggesting that these conserved miRNAs could also play an important role in the maintenance of seed maturation programmes or seed germination.

Embryo development can be generally classified into three overlapping stages, namely embryogenesis, maturation and desiccation (Goldberg et al., 1994). In a developing plant seed, following embryogenesis stage, embryo development will go through a transition period toward maturation phase when lipids and storage proteins are actively accumulated. In the late stage of seed development, the seed reaches its maximum dry weight while its water content declines. Developmental arrest and the ability to withstand desiccation of developing seeds at this stage enable them to remain in a quiescent state without undergoing precocious germination. It is well established that plant embryogenesis and seed development is a tightly regulated process and mechanisms are in place to prevent its precocious induction during early embryogenesis. miR156 is a master regulator of developmental transitions in flowering plants, and its accumulation is temporally regulated with highest expression levels occurring in early stage of embryo development (Wang et al., 2009; Tang et al., 2012). In Arabidopsis miR156 was found to play a major role in early embryo patterning and in preventing the precocious expression of maturation genes, by regulating its targets *SPL10* (*Squamosa Promoter-Binding protein-like 10*) and *SPL11* (Tang et al., 2012). It was observed that the high level expression of miR156 was maintained at late stages of seed development in Arabidopsis, but *SPL10* and *SPL11* promoter activities was up-regulated so that the transcription of *SPL10* and *SPL11* could surpass the threshold of miR156 accumulation and therefore promote maturation phase gene expression programs (Nodine and Bartel, 2010). In rice, miR159 was found to regulate some members of MYB family that play an important role in plant embryogenesis and seed development in response to the presence of abscisic acid (ABA) (Li et al., 2011b). In Arabidopsis and rice miR160 and miR167 have been identified to be involved in auxin signaling via regulation of auxin response factors (ARFs) that bind to auxin response promoter elements and mediate gene expression responses to auxin (Jones-Rhoades et al., 2006; Zhou et al., 2010). Further, miR166-mediated regulation of the type III homeodomain-leucine zipper (HD-ZIPIII) genes including *PHABULOSA* (*PHB*) and *PHAVOLUTA* (*PHV*) has also been reported to be important in the early embryonic patterning during seed development in Arabidopsis (Grigg et al., 2009). miR166 represses the expression *PHB/PHV* that promote the seed maturation program by binding to the promoter of *LEC2* that encodes a master regulator of seed development (Tang et al., 2012).

As a part of the investigation into the molecular features of HO safflower, the *ol* allele has been mapped as the defective microsomal ω-6 oleoyl phosphatidylcholine desaturase *CtFAD2-1* that is expressed specifically in the developing seed and largely responsible for the conversion of oleic acid to linoleic acid in safflower seed oil (Hamdan et al., 2012; Liu et al., 2013). In the defective *CtFAD2-1*Δ, a single nucleotide deletion in the coding region causes premature termination of translation in the HO genotype and as a result, the expression of the *CtFAD2-1*Δ was attenuated in the HO genotypes compared to the conventional HL safflower. Further study indicated that siRNAs corresponding to the *CtFAD2-1* were equally insignificant in both the HO and HL safflower genotypes, ruling out the possibility of a siRNA mediated gene expression down-regulation in the HO genotype (Liu et al., 2013). We have recently hypothesized that nonsense mediated RNA decay (NMD) is the likely underlying molecular mechanism for the HO trait in the *olol* genotype. Distinct from siRNA mediated RNA degradation mechanism, NMD may proceed through the activation of decapping and degradation in the 5′ → 3′ direction by the exoribonuclease or through the removal of the poly(A) tail from the 3′ end by deadenylase, followed by mRNA decay in the 3′ → 5′ direction performed by the exosome complex (Tomecki and Dziembowski, 2010). We have found that 13 miRNAs were differentially expressed in the HO mutant and HL wild type safflower. For example, the expression level of cti-miR5026 in HO safflower is more than 700 times higher than that in HL safflower. Consistent with the previous finding in mature safflower seed (Li et al., 2011a), miR2911 was found highly abundant in the HL genotype, but its presence was significantly reduced in the HO genotpye. Whether cti-miR5026 and cti-miR2911 are involved in the regulation of the attenuated expression of *CtFAD2-1*Δ and the NMD activation in HO safflower remains to be explored further.

To our knowledge, this work provides the first small RNA expression analysis in safflower developing embryos coinciding with the most active metabolite accumulation period for seed storage lipids and proteins. It is also the first comparative miRNA profiling analysis between two plant genotypes with significantly different fatty acid profiles. The comparative profiling of entire sets of sRNA transcriptome, especially the miRNA transcriptome, lays a foundation to a resource that could contribute to unraveling the complex miRNA-mediated regulatory networks controlling seed development and oil accumulation in safflower.

### Conflict of interest statement

The authors declare that the research was conducted in the absence of any commercial or financial relationships that could be construed as a potential conflict of interest.

## Supplementary material

Supplementary Figure S1 **Precursors and hairpin structures of known miRNAs**.

Supplementary Table S1 **A list of the primers used in stem-loop RT-PCR**.

Supplementary Table S2 **Predicted targets of safflower miRNAs**.
