# Supplementary material for: Comparative profiling of miRNA expression in developing seeds of high linoleic and high oleic safflower (Carthamus tinctorius L.) plants
Source: Front Plant Sci. 2013 Dec 2;4:489. doi: 10.3389/fpls.2013.00489 (PMC3844856; doi:10.3389/fpls.2013.00489)

A. Cti-miR156

TTGTTTGGGAGAGAGATAGAGCTGACAGAAGAGAGTGAGCACACAAAGGCAAATTATACCCAAGA
GTCTTGTATGATTGTTTTGGGTGCTCACTGCTCTATCTGTCACCTTCATCCTCCCTTT

(miRNA* found in both HL and HO libraries)

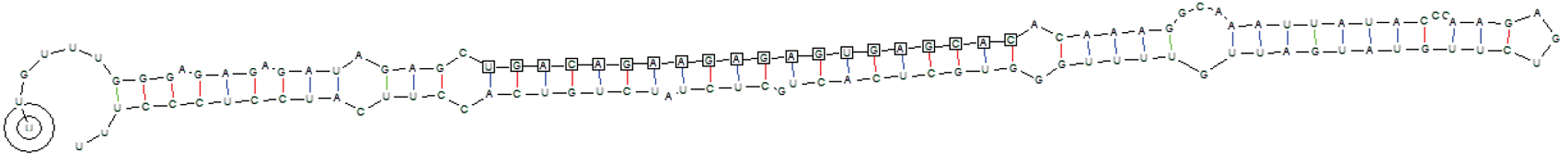

B. Cti-miR162

TGTGCGTTTGGTGGAGTTCATTGGAGTCAGCGGTTCATCGATCAGTTCCTCTAACAAACATACGAACA
AGGGGGAGAAAGAGAGAGGAATCGATCGATAAACCTCTGCATCCAGTGTCCACCCCTGCCGTGCC

(miRNA* was found in neither HL nor HO library)

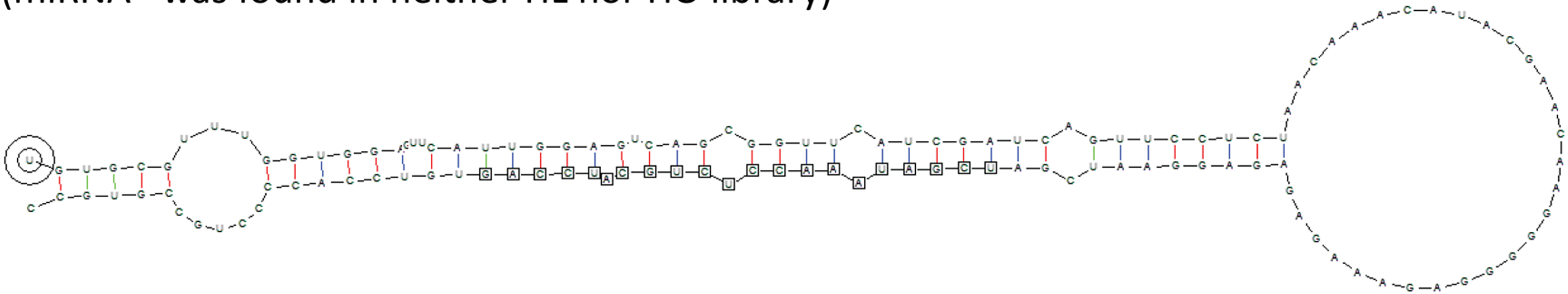

Supplement: Supplementary Figure S1 — Precursors and hairpin structures of known miRNAs. [file Presentation1.PDF]
