# Supplementary material for: Comparative profiling of miRNA expression in developing seeds of high linoleic and high oleic safflower (Carthamus tinctorius L.) plants
Source: Front Plant Sci. 2013 Dec 2;4:489. doi: 10.3389/fpls.2013.00489 (PMC3844856; doi:10.3389/fpls.2013.00489)
Supplement: Supplementary Table S1 — A list of the primers used in stem-loop RT-PCR. [file DataSheet1.DOCX]

**SUPPLEMENTARY Table 1 ⎮A list of the primers used in stem-loop RT-PCR**

| **miRNA** | **Primer** | **Sequence (5'-3')** | **Length** |
| --- | --- | --- | --- |
|  | Reverse | ATCCAGTGCAGGGTCCGAGG | 20 |
| miR156 | RT | GTCGTATCCAGTGCAGGGTCCGAGGTATTCGCACTGGATACGACGTGCTC | 50 |
|  | Forward | GCGGCGGTGACAGAAGAGAGTG | 22 |
| miR390 | RT | GTCGTATCCAGTGCAGGGTCCGAGGTATTCGCACTGGATACGACGGCGCT | 50 |
|  | Forward | GCGGCGGAAGCTCAGGAGGGATAG | 24 |
| miR157 | RT | GTCGTATCCAGTGCAGGGTCCGAGGTATTCGCACTGGATACGACGTGCTC | 50 |
|  | Forward | GCGGCGGTTGACAGAAGATAGAG | 23 |
| miR159 | RT | GTCGTATCCAGTGCAGGGTCCGAGGTATTCGCACTGGATACGACTAGAGC | 50 |
|  | Forward | GCGGCGGTTTGGATTGAAGGGAG | 23 |
| miR162 | RT | GTCGTATCCAGTGCAGGGTCCGAGGTATTCGCACTGGATACGACCTGGAT | 50 |
|  | Forward | GCGGCGGTCGATAAACCTCTGC | 22 |
| miR160 | RT | GTCGTATCCAGTGCAGGGTCCGAGGTATTCGCACTGGATACGACTGGCAT | 50 |
|  | Forward | GCGGCGGTGCCTGGCTCCCTG | 21 |
| miR172 | RT | GTCGTATCCAGTGCAGGGTCCGAGGTATTCGCACTGGATACGACATGCAG | 50 |
|  | Forward | GCGGCGGAGAATCTTGATGATG | 22 |
| miR171 | RT | GTCGTATCCAGTGCAGGGTCCGAGGTATTCGCACTGGATACGACGATATT | 50 |
|  | Forward | GCGGCGGTGATTGAGCCGTGCC | 22 |
| miR319 | RT | GTCGTATCCAGTGCAGGGTCCGAGGTATTCGCACTGGATACGACAGGGAG | 50 |
|  | Forward | GCGGCGGTTGGACTGAAGGGAG | 22 |
| miR858 | RT | GTCGTATCCAGTGCAGGGTCCGAGGTATTCGCACTGGATACGACCAAGGT | 50 |
|  | Forward | GCGGCGGTTCGTTGTCTGTTCG | 22 |
